# Long-Term Deleterious Effects of Short-term Hyperoxia on Cancer Progression—Is Brain-Derived Neurotrophic Factor an Important Mediator? An Experimental Study

**DOI:** 10.3390/cancers12030688

**Published:** 2020-03-14

**Authors:** Adrian Tiron, Irina Ristescu, Paula A. Postu, Crina E. Tiron, Florin Zugun-Eloae, Ioana Grigoras

**Affiliations:** 1 TRANSCEND Research Centre, Regional Institute of Oncology, 700483 Iasi, Romania; adrian.tiron@iroiasi.ro (A.T.); paula.postu@iroiasi.ro (P.A.P.); florin.zugun@iroiasi.ro (F.Z.-E.); 2 Department of Anaesthesia and Intensive Care, School of Medicine, “Grigore T. Popa” University of Medicine and Pharmacy, 700115 Iasi, Romania; anca.ristescu@umfiasi.ro (I.R.); ioana.grigoras@umfiasi.ro (I.G.); 3 Department of Anaesthesia and Intensive Care, Regional Institute of Oncology, 700483 Iasi, Romania; 4 Department of Immunology, School of Medicine, “Grigore T Popa” University of Medicine and Pharmacy, 700400 Iasi, Romania

**Keywords:** cancer progression, breast cancer, hyperoxia, perioperative, metastasis, BDNF, Vimentin, E-Cadherin, epithelial mesenchymal transition, angiogenesis

## Abstract

Perioperative factors promoting cancer recurrence and metastasis are under scrutiny. While oxygen toxicity is documented in several acute circumstances, its implication in tumor evolution is poorly understood. We investigated hyperoxia long-term effects on cancer progression and some underlying mechanisms using both in vitro and in vivo models of triple negative breast cancer (TNBC). We hypothesized that high oxygen exposure, even of short duration, may have long-term effects on cancer growth. Considering that hyperoxic exposure results in reactive oxygen species (ROS) formation, increased oxidative stress and increased Brain-Derived Neurotrophic Factor (BDNF) expression, BDNF may mediate hyperoxia effects offering cancer cells a survival advantage by increased angiogenesis and epithelial mesenchymal transition (EMT). Human breast epithelial MCF10A, human MDA-MB-231 and murine 4T1 TNBC were investigated in 2D in vitro system. Cells were exposed to normoxia or hyperoxia (40%, 60%, 80% O_2_) for 6 h. We evaluated ROS levels, cell viability and the expression of BDNF, HIF-1α, VEGF-R2, Vimentin and E-Cadherin by immunofluorescence. The in vivo model consisted of 4T1 inoculation in Balb/c mice and tumor resection 2 weeks after and 6 h exposure to normoxia or hyperoxia (40%, 80% O_2_). We measured lung metastases and the same molecular markers, immediately and 4 weeks after surgery. The in vitro study showed that short-term hyperoxia exposure (80% O_2_) of TNBC cells increases ROS, increases BDNF expression and that promotes EMT and angiogenesis. The in vivo data indicates that perioperative hyperoxia enhances metastatic disease and this effect could be BDNF mediated.

## 1. Introduction

Breast cancer currently represents the most prevalent malignancy and the leading cause of cancer mortality in Western women [1]. Frequently, the cause of death is not related to the primary tumor but rather to local recurrence and metastasis. Cancer evolution after radical resection depends on many factors, mainly the stage of the disease, tumor biological features, initial therapy and immune function status [2]. During the perioperative period several events, such as surgical insult, systemic inflammation, anesthetic and analgesic drugs and blood transfusion, can favor cancer progression [3,4].

Oxygen is the most common used drug in the perioperative period. General anesthesia invariably results in perioperative supplemental oxygen administration. Long-time considered as a definitely useful and harmless intervention, oxygen therapy is under scrutiny in recent years. The accumulation of data showing hyperoxia harmful effects during acute events (stroke, myocardial infarction, cardiac arrest) was followed by guidelines recommendation for oxygen use [5,6]. The optimal perioperative oxygen concentration is still poorly defined for specific circumstances. Clinical trials suggest that surgical oncological patients may have an increased risk of mortality and cancer progression when exposed to high oxygen concentration [7,8].

The main mechanism of oxygen toxicity is mediated by free radical oxygen species (ROS). ROS production may be followed by increased expression of Brain-Derived Neurotrophic Factor (BDNF) [9,10,11]. BDNF is an intracellular protein—a member of the neurotrophin family—involved in neurogenesis and neuronal differentiation [12]. In cancer BDNF has been shown to play a role in proliferation, invasion and metastasis in central nervous system, mammary, ovarian, pancreatic and uterine neoplasms [13,14,15,16]. BDNF promotes angiogenesis by increasing VEGFR expression through HIF-1α [17].

Small levels of ROS will induce insufficient BDNF expression, while high levels of ROS will induce extended BDNF stimulation leading to epithelial mesenchymal transition (EMT) and angiogenesis. EMT induces new cancer stem cell formation that may be responsible for cancer recurrence. However, very high levels of ROS induced by exposure to over 80% O_2_ are deleterious and induce apoptosis [18]. This suggests that there is a narrow window in which certain degree of hyperoxia can promote cancer progression.

We hypothesize that high concentration oxygen exposure, even of short duration, may have long term effects on cancer progression, mainly during the perioperative period when the above mentioned concurrent factors intervene. Taking into account that hyperoxic exposure results in ROS formation, increased oxidative stress and increased BDNF expression, BDNF may mediate hyperoxia effects offering cancer cell a survival advantage by several mechanisms like increased angiogenesis and EMT.

In order to test this hypothesis we designed an experimental model of breast cancer cells exposed for 6 h to various oxygen concentrations and we explored the extent of metastatic process (in vivo model) and the mechanisms involved (in vitro and in vivo models).

In vitro study hypothesis—Hyperoxia increases ROS production, amplifies BDNF expression, followed by EMT activation (increased Vimentin and decreased E-Cadherin expression) and angiogenesis (increased VEGF-R2 expression).

The objectives of this study were:

1. To identify hyperoxia effects on cell viability and ROS production

2. To evaluate the expression of molecules involved in angiogenesis and EMT in breast cancer cells.

In vivo study hypothesis—Short term perioperative hyperoxia enhances breast cancer progression and metastasis.

The objectives of the study were:

1. To evaluate lung metastasis at 4 weeks after tumor resection and oxygen exposure

2. To evaluate the expression of BDNF and molecules involved in angiogenesis and EMT.

## 2. Results

### 2.1. In Vitro Model—Breast Cancer Cell Cultures

**Figure 1:**
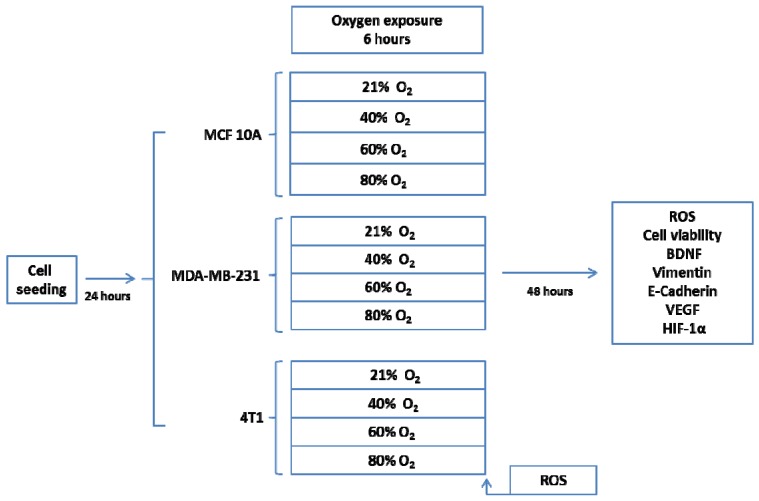
In vitro experimental protocol.

Two highly aggressive, triple negative breast cancer (TBNC) cell lines (human MDA-MB-231 and murine 4T1 cell line) were exposed to 21%, 40%, 60% and 80% O_2_ concentration for 6 h. As a control, the human epithelial breast cell line MCF10A was used. The measured parameters were ROS (oxidative stress) at the end of the exposure and 48 h later and cell viability, BDNF, E-Cadherin and Vimentin (EMT markers), VEGF-R2 (angiogenesis marker), HIF-1α at 48 h after the exposure (Figure 1).

#### 2.1.1. ROS Levels and Cell Viability

**Figure 2:**
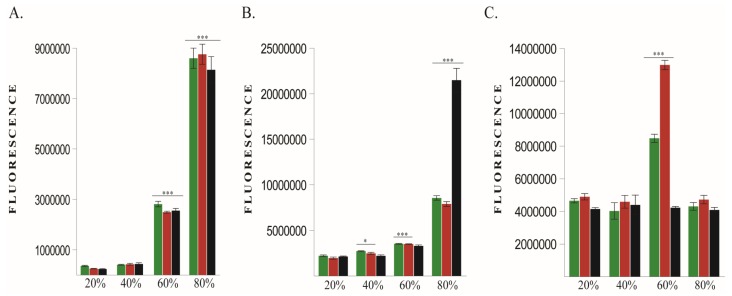
Reactive oxygen species (ROS) levels and cell viability. (**A**) ROS right after oxygen exposure; (**B**) ROS at 48h post oxygen exposure; (**C**) Cell viability. Green—MCF10A, Red—MDA-MB-231, Black—4T1. * *p* < 0.05, *** *p* < 0.0005.

Immediately after exposure all 3 cell lines showed significantly increased ROS production in 60% and 80% O_2_ groups compared to non-exposed cells (Figure 2A). At 48 h after exposure, ROS levels were still elevated in 60% and 80% O_2_ groups (Figure 2B). Interesting, at 48 h human cell lines displayed a significant increase of ROS in 40% group, although at the end of oxygen exposure ROS were not significantly increased. That means that ROS continued to increase after ending of 40% oxygen treatment.

Cell viability was increased at 48 h after exposure, in 60% O_2_ exposed human cell lines but not in murine cancer cells (Figure 2C).

Taking into consideration that 48 h post oxygen exposure ROS were still high in 80% groups especially in murine cell line and that in 60% groups only human cell lines exhibited increased viability/proliferation and considering the fact that human and murine cells may have different dynamics, we decided to evaluate proposed molecules only in human cell lines for in vitro designs and murine line in in vivo experiments.

#### 2.1.2. BDNF Expression

**Figure 3:**
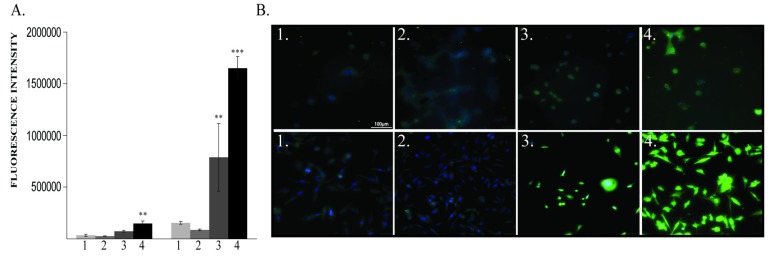
Brain-Derived Neurotrophic Factor (BDNF) protein expression. (**A**) BDNF protein quantification: left MCF10A, right MDA-MB-231; (**B**) Immunofluorescence pictures: upper panel MCF10A, lower panel MDA-MB-231; 1—21% O_2_, 2—40% O_2_, 3—60% O_2_, 4—80% O_2_. ** *p* < 0.005, *** *p* < 0.0005.

BDNF levels at 48 h after exposure to different oxygen concentrations were increased in both 60% and 80% O_2_ exposed MDA-MB-231 cells. In MCF10A cells, BDNF was significantly increased only in the 80% O_2_ group (Figure 3A,B).

#### 2.1.3. Vimentin and E-Cadherin Expression

**Figure 4:**
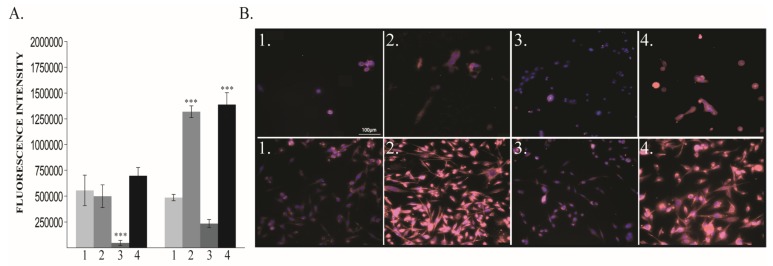
Vimentin protein expression. (**A**) Protein quantification: left MCF10A, right MDA-MB-231 (**B**) Immunofluorescence pictures: upper panel MCF10A, lower panel MDA-MB-231; 1—21% O_2_, 2—40% O_2_, 3—60% O_2_, 4—80% O_2_. *** *p* < 0.0005.

The oxygen exposure modulates EMT associated markers at 48 h, especially in human cancer cell line. In MDA-MB-231, Vimentin expression significantly increased in 40% and 80% O_2_ groups but surprisingly decreased in 60% group without being statistically significant (Figure 4A,B). Moreover, that decrease was mirrored in normal cells, where it was statistically significant. (Figure 4).

**Figure 5:**
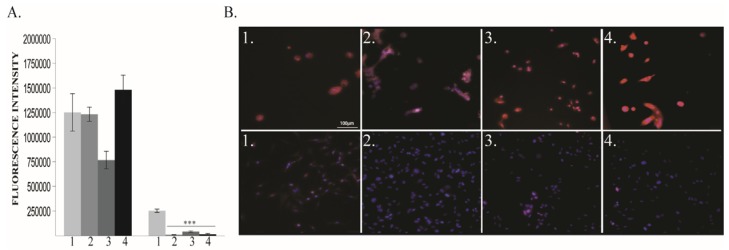
E-Cadherin protein expression. (**A**) Protein quantification: left MCF10A, right MDA-MB-231 (**B**) Immunofluorescence pictures: upper panel MCF10A, lower panel MDA-MB-231; 1—21% O_2_, 2—40% O_2_, 3—60% O_2_, 4—80% O_2_. *** *p* < 0.0005.

E-Cadherin showed similar expression pattern as Vimentin in MCF10A. However, E-Cadherin non-significantly decreased in 60% group of MCF10A, and, as expected, significantly decreased in all treated groups of MDA-MB-231 cells (Figure 5A,B).

#### 2.1.4. VEGF-R2 Expression

**Figure 6:**
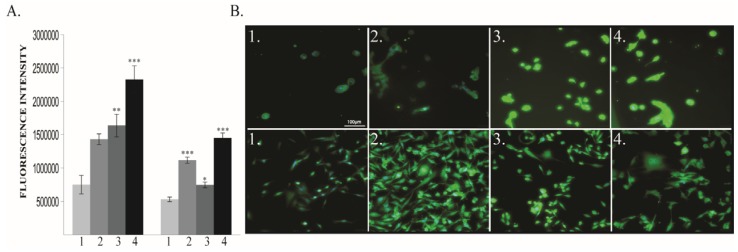
VEGF-R2 protein expression. (**A**) Protein quantification: left MCF10A, right MDA-MB-231; (**B**) Immunofluorescence pictures: upper panel MCF10A, lower panel MDA-MB-231; 1—21% O_2_, 2—40% O_2_, 3—60% O_2_, 4—80% O_2_. * *p* < 0.05, ** *p* < 0.005, *** *p* < 0.0005.

VEGF-R2 expression has been found to be dependent on oxygen treatment in both human cell lines (Figure 6A,B).

In normal cells, VEGF R2 expression increased in a dose-dependent manner. In MDA-MB-231 cells, although in all treated groups it was significantly increased, 60% O_2_ group has a lower expression compared to 40% and 80% O_2_ group.

#### 2.1.5. HIF-1α Expression

**Figure 7:**
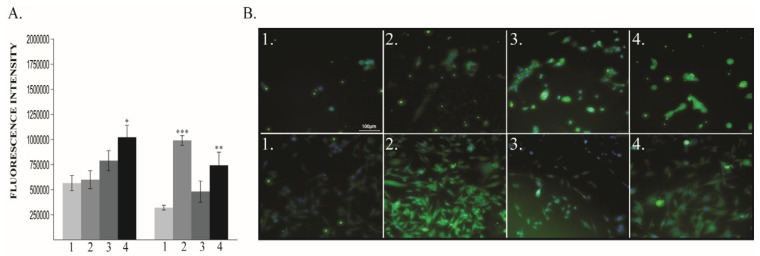
HIF-1α protein expression. (**A**) Protein quantification: left MCF10A, right MDA-MB-231; (**B**) Immunofluorescence pictures: upper panel MCF10A, lower panel MDA-MB-231; 1—21% O_2_, 2—40% O_2_, 3—60% O_2_, 4—80% O_2_. * *p* < 0.05, ** *p* < 0.005, *** *p* < 0.0005.

HIF-1α displayed significantly increased expression in 40% and 80% oxygen treated groups in MDA-MB-231 cells, mainly in the 40% group (Figure 7A,B). In MCF10A, HIF-1α significantly increased only in 80% O_2_ groups.

The results of the in vitro experiment are summarized in Table 1.

### 2.2. In vivo Model—Murine Breast Cancer

**Figure 8:**
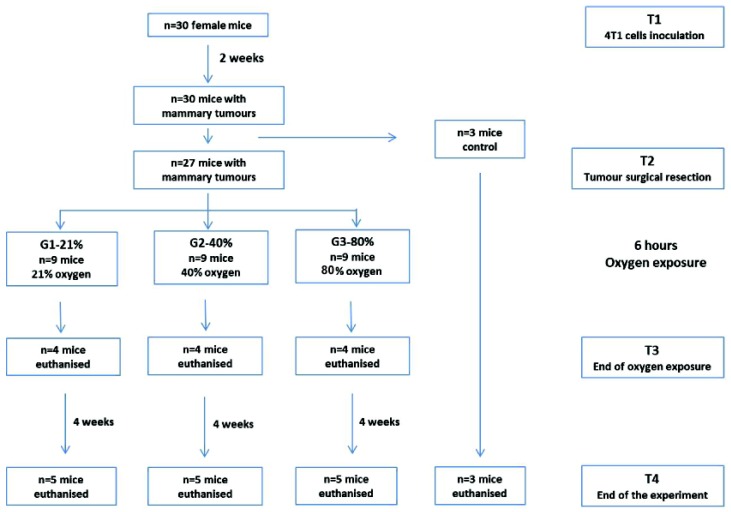
In vivo experimental protocol.

Murine TNBC 4T1 cells were inoculated into the mammary gland of 8 weeks old females BALB/c mice. After 2 weeks, the primary tumor was surgically removed under general anesthesia. Animals were randomly allocated into 3 groups: G1-21%—mice perioperatively exposed to 21% O_2_ (atmospheric air), G2-40%—mice perioperatively exposed to 40% O_2_ and G3-80%—mice perioperatively exposed to 80% O_2_. The duration of oxygen exposure was 6 h, considered the mean time period for perioperative oxygen administration in surgical patients. Mice from each group were sacrificed after the oxygen exposure (n = 4) and at 4 weeks after surgery (n = 5). Lungs were in vitro evaluated for the presence and development of metastases together with the expression of BDNF, E-Cadherin, Vimentin, VEGF-R2 and HIF-1α (Figure 8).

#### 2.2.1. Lung Metastasis

**Figure 9:**
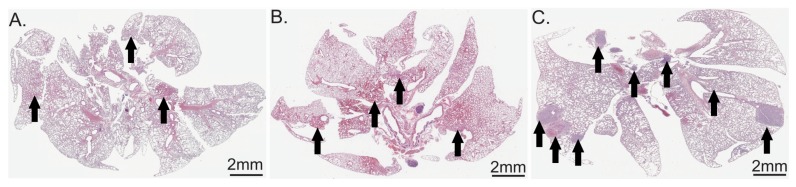
Hematoxylin and eosin staining of lungs. Arrows indicate metastases. (**A**) G1-21% oxygen; (**B**) G2-40% oxygen; (**C**) G3-80% oxygen.

At 4 weeks after surgery and oxygen exposure (T4) lung specimens from G1-21%, G2-40% and G3-80% groups were submitted to hematoxylin eosin staining and optic microscopy examination. The examination revealed increased metastasis number and size in all O_2_ exposed groups, the most striking ones being present in the G3-80% group (Figure 9).

#### 2.2.2. BDNF Expression

**Figure 10:**
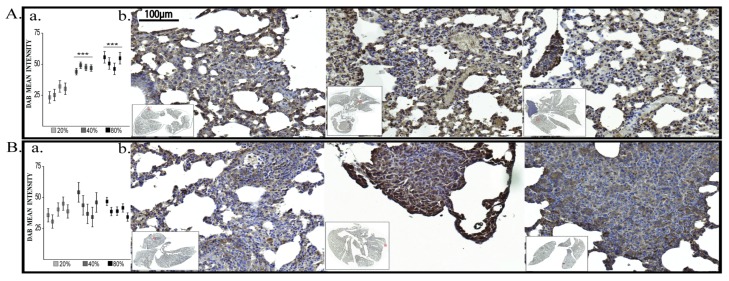
In vivo BDNF protein expression. (**A**) At the end of oxygen exposure (T3) (**B**) At the end of experiment (T4); a. Protein quantification, each data point represent one mouse b. IHC pictures: left G1-21%, middle G2-40%, right G3-80%; Immunofluorescence pictures; *** *p* < 0.0005.

At the end of oxygen exposure (T3), BDNF expression showed significant increase in both G2-40% and G3-80% groups (Figure 10A).

This data demonstrate the existence of a BDNF synthesis peak in G2-40% group but smaller then in G3-80% group. In addition, although not all cells present increased BDNF expression, the responsive cells shifted BDNF expression from mainly cytoplasmic to nuclear or perinuclear expression. However, at 4 weeks after exposure (T4) there were no differences of BDNF expression across the investigated groups (Figure 10B).

#### 2.2.3. Vimentin Expression

**Figure 11:**
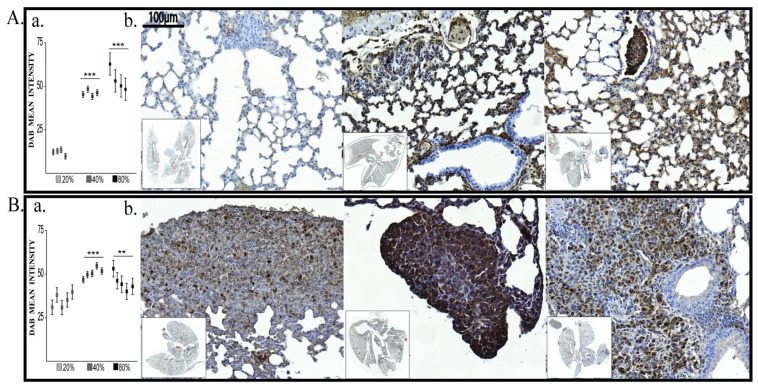
In vivo Vimentin protein expression. (**A**) At the end of oxygen exposure (T3) (**B**) At the end of experiment (T4); a. Protein quantification, each data point represent one mouse b. IHC pictures: left G1-21%, middle G2-40%, right G3-80%; Immunofluorescence pictures: ** *p* < 0.005, *** *p* < 0.0005.

At the end of oxygen exposure (T3) only Vimentin was significantly elevated in G2-40% and G3-80% groups compared to G1-21% group. Moreover, we found Vimentin significantly elevated also at T4 (the end of experiment) (Figure 11A,B).

#### 2.2.4. E-Cadherin, VEGF-R2 and HIF-1α Evaluation

Other molecules (E-Cadherin, VEGF-R2 and HIF-1α) have not been yet modulated by oxygen immediately after exposure (Figure S1).

## 3. Discussion

In this experimental study, we investigated long-term effects of short-term hyperoxia on cancer progression and some underlying mechanisms using both in vitro and in vivo models of triple negative breast cancer. Compared to normoxia group, perioperative administration of 80% oxygen for 6 h in a murine TNBC model increased the size and number of lung metastasis at 4 weeks after surgery, along with increased BDNF and Vimentin expression at the end of oxygen exposure.

In clinical setting, perioperative administration of high inspiratory oxygen levels is currently used to avoid hypoxemia and tissue hypoxia. The accumulation of data on hyperoxia harmful effects in the acute settings [19,20] raised the question about the optimal oxygen concentration to avoid postoperative short-term complications, in the general surgical population or in specific subgroups [5,6].

In cancer patients, beside short-term consequences, hyperoxia effects on long-term cancer evolution are under scrutiny. Oxygen administration of 80% during the perioperative period in abdominal surgery was associated with increased long-term mortality, statistically significant in cancer patients comparing with non-cancer patients [7]. The risk of new or recurrent cancer after high (80%) versus normal (21%) inspiratory oxygen during abdominal surgery was assessed in 1377 patients, who completed a median 3.9 years follow-up period. The study showed that new cancers occurred at similar rate but the cancer-free survival time was significantly shorter in the 80% oxygen group [8]. While hyper/normobaric hyperoxia was suggested to enhance chemo- and radiotherapy in certain cancer types or to have antitumoral effects [21,22,23], normobaric hyperoxia during/after surgery is poorly investigated and its effects on cancer progression are sparse. For the moment, no pathway was described to link these two.

Hyperoxia never occurs during natural circumstances. Thus, while normal tissues and cells have extensive adaptive mechanisms to hypoxemia and hypoxia, they have limited protection from hyperoxia [24]. High concentration oxygen exposure results in increased ROS formation and oxidative stress, DNA damage, protein damage and lipid peroxidation [25]. The severity of cell disturbances depends on the degree and duration of exposure and on cell susceptibility. Thus, oxidative stress may lead to cell damage or cell death by apoptosis or necrosis. At the same time, when a cell experiences high stress levels, it activates autocrine/paracrine mechanisms to protect itself and other cells. These subtle effects are almost silent and undetected during the postoperative recovery but may have long-term outcome consequences.

As far as we know, this is the first experimental study investigating hyperoxia long-term effects on a surgical cancer model.

### 3.1. BDNF Expression is Enhanced by 6 h O_2_ Exposure in Triple Negative Breast Cancer Cells

BDNF acts as an antioxidant to protect neuronal cells against oxidative stress [9] and attenuates oxidative stress in cancer cells. It was shown that in pheocromocytoma (PC12) cells, subtoxic levels of ROS induced BDNF expression, which may have a protective role, since BDNF pre-treatment significantly attenuated PC12 cell death [26]. BDNF can induce cancer cell survival, proliferation, resistance to anoikis and angiogenesis. The tumor cells have the ability to alter the local balance between pro- and anti-angiogenetic factors, favoring the former. It was proven that BDNF promotes the expression of a strong pro-angiogenetic factor, VEGF via HIF-1α in neuroblastoma cells [11]. The expression of BDNF, its receptor and a high degree of vascularization are poor prognosis factors in neuroblastoma [27]. BDNF can be produced by neurons, astrocytes, macrophages, fibroblasts and B lymphocytes and cancer cells, and exerts its effect mainly by acting on its receptors—with high affinity to tyrosine receptor kinase B (TrkB) and with low affinity on p75 receptor. It has been shown that TrkB positive cancer stem cells contribute to recurrence of TNBC in a mouse model [28] while p75 receptor expression is a characteristic of mitotically quiescent cancer stem cell population which enhances their chemoresistance [29].

In our in vitro model, BDNF expression increased in breast cancer cells after exposure to 60% and 80% O_2_ but did not increase in 40% O_2_ group, probably related to lower ROS increment right after exposure. This time point may not be the optimal one to measure BDNF dynamics in 40% O_2_ group but increased Vimentin and decreased E-Cadherin expression in cancer cell were already present to demonstrate a pick of undetected BDNF expression. After 80% oxygen exposure, some MDA-MB-231 cells displayed typical mesenchymal shape but some others may enter into apoptosis as they presented bleb formation, as shown by others [30].

The in vivo model showed that immediately after 40% and 80% O_2_ exposure, BDNF starts to induce EMT as Vimentin increased, while E-Cadherin did not decrease yet. We can conclude that increased BDNF expression is a result of high oxygen administration and it triggers several events that ultimately lead to increased metastasis. HIF-1α and VEGF induction may be slower since they were not detected at this time point.

### 3.2. EMT is Enhanced by 6 h O_2_ Exposure in Triple Negative Breast Cancer Cells

EMT is a biological process that allows a polarized epithelial cell, in contact with the basement membrane, to switch to a mesenchymal phenotype and migrates away from the original epithelial layer. EMT is an extremely important cellular program in embryogenesis and tissue healing. In cancer, the onset of EMT is associated with increased metastasis potential [31]. The markers used to highlight EMT are increased Vimentin and decrease E-Cadherin expression. Vimentin is a cytoskeleton protein associated with a migration phenotype. High Vimentin expression represents a poor prognostic marker in cancer [32,33,34]. E-Cadherin is a cytoplasmic protein present in the epithelial cells. Its reduced expression in the mesenchymal cell correlates with an invasive phenotype [35].

In cancer, EMT is considered a driver of metastasis [36]. Due to the fact that EMT is also correlated with cancer stem cell generation that will lead to drug resistance and tumor recurrence, we considered to investigate EMT in the context of hyperoxia. Our in vitro study indicates that Vimentin was upregulated in 40% and 80% oxygen exposed TNBC cells. The decreased levels of Vimentin (decrease that is not statistically significant) and E-Cadherin in 60% O_2_ group may be correlated with increased proliferation, suggesting that this oxygen regimen induces an increased proliferation state in some cells and EMT in others. While total cell immunofluorescence analysis of 60% group showed a small decrease of Vimentin expression instead of the expected increase, acquired pictures still show the presence of cells with high Vimentin expression, among other cells with low expression. The high Vimentin expression cells indicate ongoing EMT; the low Vimentin expression cells may be instead in proliferative states. Overall E-Cadherin dropped in 60% group although not at the values recorded in 40% and 80% groups. This may reflect again the fact that some cells entered EMT (decreasing E-Cadherin) while other cells did not enter in a full EMT. Since we have a single static point for immunofluorescence staining, 48 h post exposure, we do not know the detailed dynamic of Vimentin and E-Cadherin. In addition, recent evidences suggest that EMT is a spectrum [36] of cells somewhere between fully epithelial and fully mesenchymal. In partial EMT, cells co-express epithelial and mesenchymal markers or will lose epithelial markers without gain of mesenchymal markers [36]. That may be also the case of many cells from 60% group which presented loss of E-Cadherin without gaining overexpression of Vimentin. Partial EMT may confer tumor cells enhanced plasticity that is crucial for metastasis [36]. Modulation of Vimentin and E-Cadherin in the 40% and 80% O_2_ groups demonstrates that BDNF induces EMT in MDA-MB-231 cell line but not in normal ones. In our setup, acute hyperoxia induces EMT. By contrast, hyperbaric hyperoxia administered 90 min/day for four days has opposite effect, inducing mesenchymal-epithelial transition. The opposite results can be explained by the experimental design differences: normobaric versus hyperbaric, 40-80% versus 100% oxygen and single 6 h exposure versus 4 sessions of 90 min each [37].

In another set of evidences, Crowley and co-authors [38] investigated the effects of 30%, 60% and 80%, 3 h oxygen exposure on two cancer cell lines, MCF-7 and MDA-MB-231. Viability was not significantly changed in any cell type and treatment regimen at 24 h post exposure and authors did not report cell death in 80% group. Our data, recorded at 48 h, after a double time interval of oxygen administration do not show changed viability, excepting human cell lines at 60% group. This effect may be explained by differences in exposure time and in post exposure time of viability measurement. The data of both studies, despite some design differences, are in line with each other and both do not find significant viability changes at 80% oxygen. Altogether, Crowley’s reported data and our current data demonstrate that not always a massive cell apoptosis upon high oxygen treatment exists. Moreover, Crowley and co-authors reported increased cell migration in 30% and 60% groups and these results are similar to our data. More importantly, they investigated migration, while we investigate EMT. Migration and invasion, although share some common mechanisms, also present some particularities, as migration consists in movement of cells, while invasion, in addition to migration, requires active degradation of a matrix in in vitro assays. Usually, migration is measured at 24 h, while invasion at 48 h. The differences between 80% groups may be explained by occurrence of full EMT, which requires some time to be achieved. Twenty-four hours post oxygen exposure there is no increase in cell migration but at 48 h there is a significant expression of EMT markers.

### 3.3. Angiogenesis is Enhanced by 6 h O_2_ Exposure in Triple Negative Breast Cancer Cells

Angiogenesis is an essential factor for tumor development and metastasis. Our in vitro data showed increased VEGF in all hyperoxia exposed breast cancer cell groups. This result is in line with previous published data reporting increased angiogenetic factors (including VEGF) in MDA-MB-231 cells exposed to high fractional oxygen [38].

We also identified that HIF-1α can be upregulated in TNBC cells after short-term hyperoxia exposure. There is a vast body of literature demonstrating that HIF-1α increases in hypoxia while in hyperoxic regimens usually decreases [17,18]. However, in skeletal muscle cells under high oxygen concentrations (42%), the amount of HSP90–HIF-1α complex is increased and suggests that HSP90 stabilizes cytoplasmic HIF-1α under high oxygen levels [39]. Moreover, in mild hyperoxia (30%) but for extended exposure (28 days) hyperoxic LNCaP xenografts displayed higher levels of VEGF and VEGF-R2 with respect to normoxic group and HIF-1α protein at higher levels than hypoxic one [40]. Even more, Xia and collaborators demonstrates that H_2_O_2_ treatment of ovarian cancer cells OVCAR-3 greatly increased HIF-1α expression up to 6h, whereas the HIF-1α-1β level was not changed [41]. Chemically, within biological tissues, oxygen may interact with H_2_O to generate H_2_O_2_, increase ROS and induce BDNF protein expression. That raises the possibility that certain degrees of hyperoxia may induce HIF-1α expression. Our data show a higher expression on 40% vs. 80% vs. 21% cancer cell groups.

In our in vivo model, although immediately after oxygen exposure we did not find higher HIF-1α in lung metastases, the pathway BDNF-HIF-1α in triggering angiogenesis via VEGF is not excluded, since it might display a slower dynamics. Our results are in line with previously reported data showing that BDNF could indeed promote the expression of VEGF via HIF-1α [27].

### 3.4. Hyperoxia Effects are Regimen-Dependent

The comparison of different oxygen concentration exposure shows that effects varied along the dose domain, with maximal effect present after 80% oxygen administration. While all hyperoxic regimens associated ROS generation, transient or persistent BDNF induction and escalating EMT, angiogenesis and metastatic tumor burden, the temporal dose-related dynamics of these phenomena is not a linear one and other concurrent mechanisms may intervene at different concentration spoiling linearity. The fact that in vivo cancer development of 40% oxygen group resemble more to 20% group, suggests that EMT induced by 80% oxygen administration represent a complete EMT while 40% oxygen induces a partial EMT.

More importantly, a very recent paper [42] demonstrates that hyperbaric oxygen (HBO) treatment promotes early recovery from muscle injury via HIF-1α protein and VEGF. HBO promoted blood vessels formation and muscle regeneration. The new paper demonstrates the beneficial role of 100% oxygen concentration (higher than we used) and authors did not report that high ROS induced by HBO result in cell apoptosis. They reported increased HIF-1α protein expression, while many other investigators reported decreased HIF-1α protein expression after 100% oxygen treatment. They did not include a cancer model to investigate side effects of HBO.

**Figure 12:**
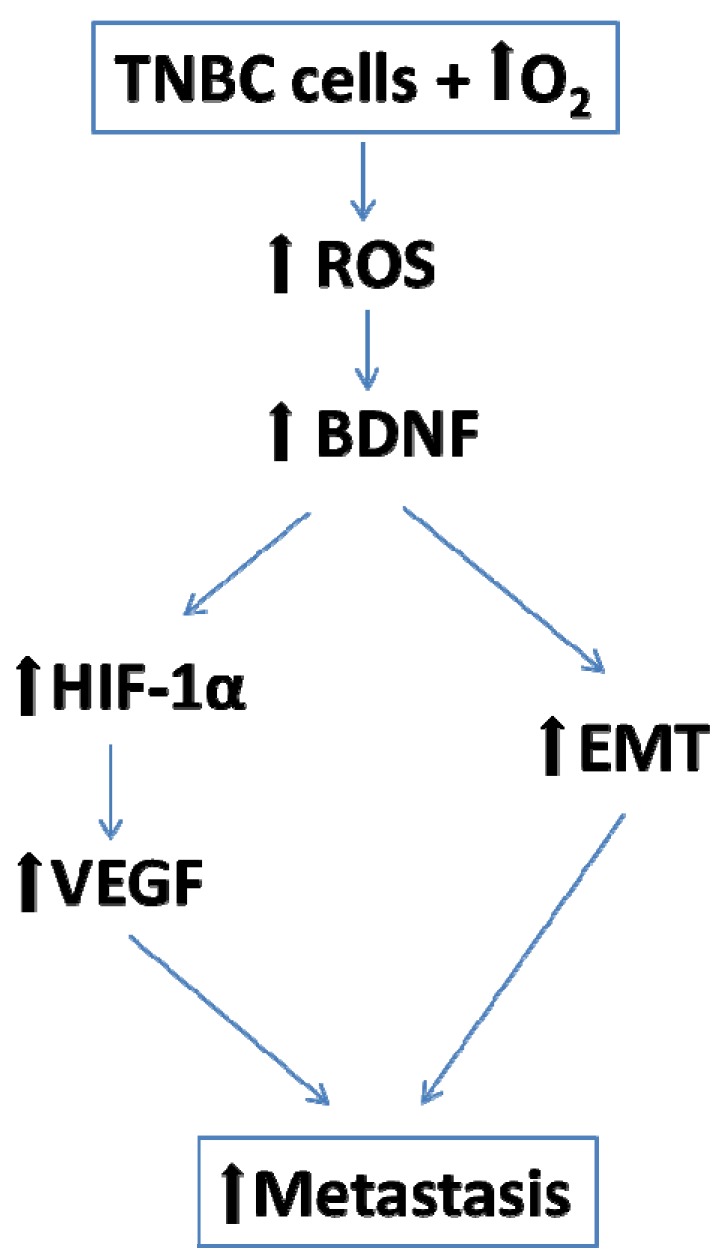
The potential role of BDNF in mediating cancer progression.

We consider all the above data highly supportive for an important role of BDNF in mediating long-term hyperoxia-related potentiation of metastatic risk at least in mammary triple negative cancer, according to the following scenario: hyperoxia—ROS—BDNF—EMT—VEGF—HIF-1α (Figure 12).

This scenario may reasonably explain HIF-1α paradoxical behavior and involvement at both extremes of oxygen exposure spectrum.

### 3.5. Study Limitations

The present study focused on the evaluation of hyperoxia effects on TNBC cells without exploring the consequences on the tumor microenvironment or the host response.

Although we show that short-term hyperoxia exposure increased the number and size of lung metastasis and that BDNF is a key mediator of this process, we did not completely prove and quantify BDNF contribution. That would require pharmacological manipulation of BDNF-TrkB axis. We have decided to assess ROS, viability and markers at 48 h but other time points may be of interest. We chose that time point due to a previous report [43] which indicates that a 48 h continuous stimulation with recombinant BDNF is required to induce EMT. EMT was abolished within 3 days after removal of 48 h BDNF stimulation. However, analysis at different time points post oxygen exposure will improve our knowledge about effects of hyperoxia.

We also did not investigate hyperoxia impact on other cancer types.

## 4. Materials and Methods

### 4.1. In vitro Model—Breast Cancer Cell Cultures

#### 4.1.1. Experimental Design

The highly aggressive human MDA-MB-231 and murine 4T1 TNBC cell lines were exposed to 21%, 40%, 60% and 80% oxygen concentration for 6 h. As control, the human epithelial breast cell line MCF10A was used. The measured parameters were ROS (oxidative stress) at the end of the exposure and after 48 h and cell viability, BDNF, HIF-1α, VEGF-R (angiogenesis marker), E-Cadherin and Vimentin (EMT markers) at 48 h after the exposure (Figure 1).

#### 4.1.2. Cell Lines and Cultures

Three cell lines were investigated: human breast epithelial MCF10A, triple negative human breast cancer MDA-MB-231 and triple negative murine mammary cancer 4T1. MCF10A, MDA-MB-231 and 4T1cells (ATCC^®^, Rockville, MD, USA) and were available at Regional Institute of Oncology’s biobank; cells were cultured in RPMI-1640 media supplemented with 10% fetal bovine serum (Sigma-Aldrich^®^, USA origin). Two thousand cells per well were seeded in 96-well flat bottom tissue culture plate in 2D culture system at 37 °C under 5% CO_2_ overnight. In each plate the 3 cell lines were seeded in equal number of wells.

#### 4.1.3. Oxygen Treatment

After 24 h since seeding, cells were exposed to 21%, 40%, 60% and 80% O_2_ for 6 h, one plate for each O_2_ concentration. During the exposure, plates were incubated in the incubation chamber of Zeiss Axio Observer Z1 TissueGnostic (Zeiss^®^, Vienna, Austria, Europe). After the oxygen exposure, cells were incubated further for 48 h.

#### 4.1.4. Fluorescence Measurements

At the end of and 48 h post O_2_ exposure, ROS were measured in 7 wells for each cell line and each O_2_ concentration. Measurement was performed in a multiplate reader (FilterMax^®^ F5, Sunnyvale, CA, USA) using dichlorofluorescein diacetate (DCFDA) (Sigma-Aldrich^®^) according to manufacturer recommendations. Cell viability was measured at 48 h after exposure in 7 wells for each cell line and O_2_ treatment regimen by microplate reader using CellTiter-Blue^®^ Cell Viability Assay (Promega^®^, Madison, WI, USA). The remaining wells belonging to each cell line and O_2_ regimen were fixed with 4% paraformaldehyde for immunofluorescence staining.

#### 4.1.5. Immunofluorescence Staining

After permeabilization with 0.3% Saponin (55825-100GM, Merck Millipore^®^, Darmstadt, Germany) and 0.3% Triton (Sigma-Aldrich^®^) for 1 h, cells were incubated at 4 °C for 72 h with primary antibodies anti-BDNF (ab108319, Abcam^®^, Cambridge, United Kingdom), 1:50; anti-Vimentin clone V9 (M0725, Dako^®^, Denmark), 1:25; anti-E-Cadherin (sc-8426, Santa Cruz Biotechnology^®^, Dallas, TX, USA), 1:25; anti-VEGF Receptor 2 (D5B1) (#9698, Cell Signaling Technology^®^, Danvers, MA, USA),1:50 and anti-HIF-1α (ab179483, Abcam^®^, Cambridge, United Kingdom),1:25. FITC conjugated goat anti-rabbit IgG secondary antibodies (F2765, ThermoFisher Scientific^®^, Waltham, MA, USA), 1:200; and Alexa Fluor 647-conjugated goat anti-mouse IgG (A21241, ThermoFisher Scientific^®^), 1:200 were applied overnight at 4 °C to respectively recognize the rabbit and the mouse IgGs. HIF-1α was multiplexed with Vimentin and VEGF-R with E-Cadherin. Fluorescent dyes were protected by using ProLong Gold Antifade Mountant with DAPI (P36941, ThermoFisher Scientific^®^) according to the manufacturer’s recommendations. Pictures were acquired at 20x with Zeiss Axio Observer Z1 Microscope TissueGnostic using Tissue FAXS 4.2 software. TissueQuest 6.0 software provided by TissueGnostic (Vienna, Austria, Europe) was used for quantitative cell analysis and to quantify sum intensity of florescence signal for each event. Nuclei were used to identify each cell in order to make quantitative analysis.

### 4.2. In Vivo Model—Murine Breast Cancer

The experiments were approved by the Ethical Committees for Scientific Research of the “Grigore T. Popa” University of Medicine and Pharmacy (38/12.02.2018) and of the Regional Institute of Oncology (10/16.01.2018), Iasi, Romania and were performed in accordance with the European legislation on the protection of animals used for scientific purpose transposed into a national law (43/2014) and with authorization from the National Sanitary Veterinary and Food Safety Authority (no. 13/04.10.2018).

#### 4.2.1. Tumor Model

The BALB/c 4T1 murine mammary cancer model was chosen as it represents one of a few breast cancer models to efficiently metastasize to sites affected in human breast cancer. Transcriptome analysis of mouse and human mammary epithelial subpopulations showed conserved gene signatures and pathways between those two species, underscoring the mouse as a viable model to study mammary development and oncogenesis [44]. Moreover, 4T1 cell line was derived from a spontaneously arising BALB/c murine mammary tumor and represents an excellent model for the study of metastatic progression in breast cancer. In addition, we decided to use a murine cell line, instead of a xenogeneic model (a human cell line injected in a mouse that lacks the immune system), because we wanted to preserve the activity of the immune system.

#### 4.2.2. Tested Animals

Female BALB/c mice, 8–10 weeks old (Cantacuzino Institute^®^, Bucharest, Romania) were used. The mice were housed in the animal facility of the Advanced Centre for Research and Development in Experimental Medicine (CEMEX), “Grigore T. Popa” University of Medicine and Pharmacy, Iasi, in individually ventilated cages (IVCs), in a climate-controlled: 20 ± 4 °C, with 50 ± 5% relative humidity and 12 h light/dark cycles, containing shaving bedding material, with regular rodent chow and water *ad libitum*.

#### 4.2.3. Tumor Engraftment and Monitoring

The inoculation procedure was performed by a standard protocol [45]. The time point of tumor cell inoculation was termed T1.

A number of 1 × 10^6^ 4T1cells suspended in 50 μL MEM-EBSS medium Matrigel (1:1) was injected into the mammary fat pad. The development of the primary tumor was daily monitored by palpation.

#### 4.2.4. Tumor Surgical Resection and Oxygen Exposure

At two weeks after 4T1 cells engraftment, all mice developed palpable mammary tumors of 8-12 mm diameter and were submitted to surgical resection under general inhalation anesthesia with Sevoflurane 2%–3%, according to standard protocols [46].

Oxygen exposure was performed during and after tumor resection for 6 h under normobaric conditions. Mice were exposed to 40% and 80% O_2_ delivered at a constant flow, 2 L per minute into the cage substrate. The time point of tumor resection and start of O_2_ exposure was termed T2.

Mice were randomly allocated into 3 equal groups of 9 mice each (Figure 8):

Group 1 (G1-21%)—mice submitted to surgical resection and perioperative exposure to 21% oxygen (atmospheric air);

Group 2 (G2-40%)—mice submitted to surgical resection and perioperative exposure to 40% oxygen;

Group 3 (G3-80%)—mice submitted to surgical resection and perioperative exposure to 80% oxygen.

The time point of end of O_2_ exposure was termed T3. At this time point four mice from each group were sacrificed.

#### 4.2.5. Tissue Collection

At 6 weeks after tumor cells engraftment and at 4 weeks after tumor resection the remaining mice (n = 5) in all groups were sacrificed by cervical dislocation. The time point of animal euthanasia was termed T4. This time point was imposed by ethical regulations for animal experiments (G3-80% animals were in severe sufferance).

#### 4.2.6. Studies in Sacrificed Animals

In all sacrificed animals (at T3 and T4 study timeline) lungs were retrieved and preserved in 10% paraformaldehyde (Sigma-Aldrich^®^). We focused on lung analysis, as it represent primary metastatic target of 4T1 cell line and because is easier to identify metastases at incipient phases. Moreover, in contrast to excised primary tumors that have not been exposed to various oxygen concentrations, lung can be investigated at both T3 and T4 time points.

#### 4.2.7. Immunohistochemistry (IHC)

IHC was performed on 4 µm thick sections of formalin fixed and paraffin embedded tissues. Sections were deparaffinized and rehydrated. Epitope recovery was performed by heating until reaching boiling point at 360 W and then maintained up to 20 min at 180 W in target retrieval solution buffer (pH 6,0 for Vimentin, E-Cadherin, VEGF Receptor 2 and pH 8.2 for BDNF and HIF-1α) using a microwave oven. Endogenous peroxidase activity was blocked by using peroxidase blocking solution (Dako, S202386-2) for 15 min in the dark at room temperature. The slides were incubated over night at 4 °C with primary antibodies diluted in antibody diluent (Dako, K800621-2): anti-E-Cadherin (Cell Signaling, 24E10), 1:200; anti-VEGF Receptor 2 (Cell Signaling, D5B1), 1:800; anti-Vimentin (Cell Signaling, D21H3), 1:100; anti-BDNF (Abcam, EPR1292), 1:600 and anti-HIF-1α (Abcam, EPR16897), 1:600. The incubations of the rabbit secondary antibody (Dako, K800921-2) and of the streptavidin— horseradish peroxidase from Dako were performed at room temperature for 30 min each. After incubation with 3,3′-diaminobezidine (Dako, Denmark) slides were counter-stained with Hematoxylin (ThermoScientific, 7211). TissueFAXS 4.2 software was used to acquire serial pictures and rebuild stained sections in digital format. Analysis for investigated markers expression was done using HistoQuest 6.0 software provided by TissueGnostic (Vienna, Austria, Europe). In tumor markers were investigated at the invasion sites and in lungs at the metastases.

### 4.3. Statistics

GraphPad Prism (La Jolla, CA, USA) software was used for statistical analysis. Grouped analyses were performed using one-way ANOVA. Quantitative data for statistical analysis were expressed as mean ± SEM (shown as error bar). Figure 10 and Figure 11 represent quantification for each mouse ± standard deviation. Significance was established when *p* < 0.05. Power analysis (InStat, La Jolla, CA, USA) showed that n = 8 animals for in vivo studies were needed to achieve a power of 0.85 with the probability of 0.05. In order to compensate for unexpected reactions of animals we used a total of 9 animals per group to achieve statistical significance.

## 5. Conclusions

We investigated the long-term effects of short-term hyperoxia on triple negative breast cancer progression and some of the underlying mechanisms. Our in vitro study showed that short-term hyperoxia exposure of breast cancer cells increases ROS, increased BDNF expression that promotes EMT and angiogenesis. The in vivo data indicates that perioperative hyperoxia enhances metastatic disease and this effect could be BDNF mediated.

## Figures and Tables

**Table 1:** The statistical significance of parameters variation in human breast cancer (MDA-MB–231) and normal epithelial (MCF 10A) cell lines exposed to 40%, 60%, 80% O_2_ (reference values-21% O_2_ exposure).

	MDA-MB–231			MCF 10A		
	40% O_2_	60% O_2_	80% O_2_	40% O_2_	60% O_2_	80% O_2_
ROS_,_ after exposure	↔	↑↑↑	↑↑↑	↔	↑↑↑	↑↑↑
ROS at 48h	↑	↑↑↑	↑↑↑	↑	↑↑↑	↑↑↑
Cell viability	↔	↑↑↑	↔	↔	↑↑↑	↔
BDNF	↔	↑↑	↑↑↑	↔	↔	↑↑↑
Vimentin	↑↑↑	↔	↑↑↑	↔	↓↓↓	↔
E-Cadherin	↓↓↓	↓↓↓	↓↓↓	↔	↔	↔
VEGF-R	↑↑↑	↑	↑↑↑	↔	↑↑	↑↑↑
HIF-1α	↑↑↑	↑	↑↑	↔	↔	↑

↔ = no statistical significance, ↑ = *p* < 0.05, ↑↑ = *p* < 0.005, ↑↑↑ = *p* < 0.0005.

## Supplementary Materials

The following are available online at https://www.mdpi.com/2072-6694/12/3/688/s1, Figure S1: IHC stainings for E-Cadherin, VEGF-R2 and HIF-1 alpha at the end of oxygen exposure.
