# Supplementary material for: Long-Term Deleterious Effects of Short-term Hyperoxia on Cancer Progression—Is Brain-Derived Neurotrophic Factor an Important Mediator? An Experimental Study
Source: Cancers (Basel). 2020 Mar 14;12(3):688. doi: 10.3390/cancers12030688 (PMC7140073; doi:10.3390/cancers12030688)

Supplimentary Figure 1. IHC stainings for E-Cadherin, VEGF-R2 and HIF-1 alpha at the end of oxygen exposure

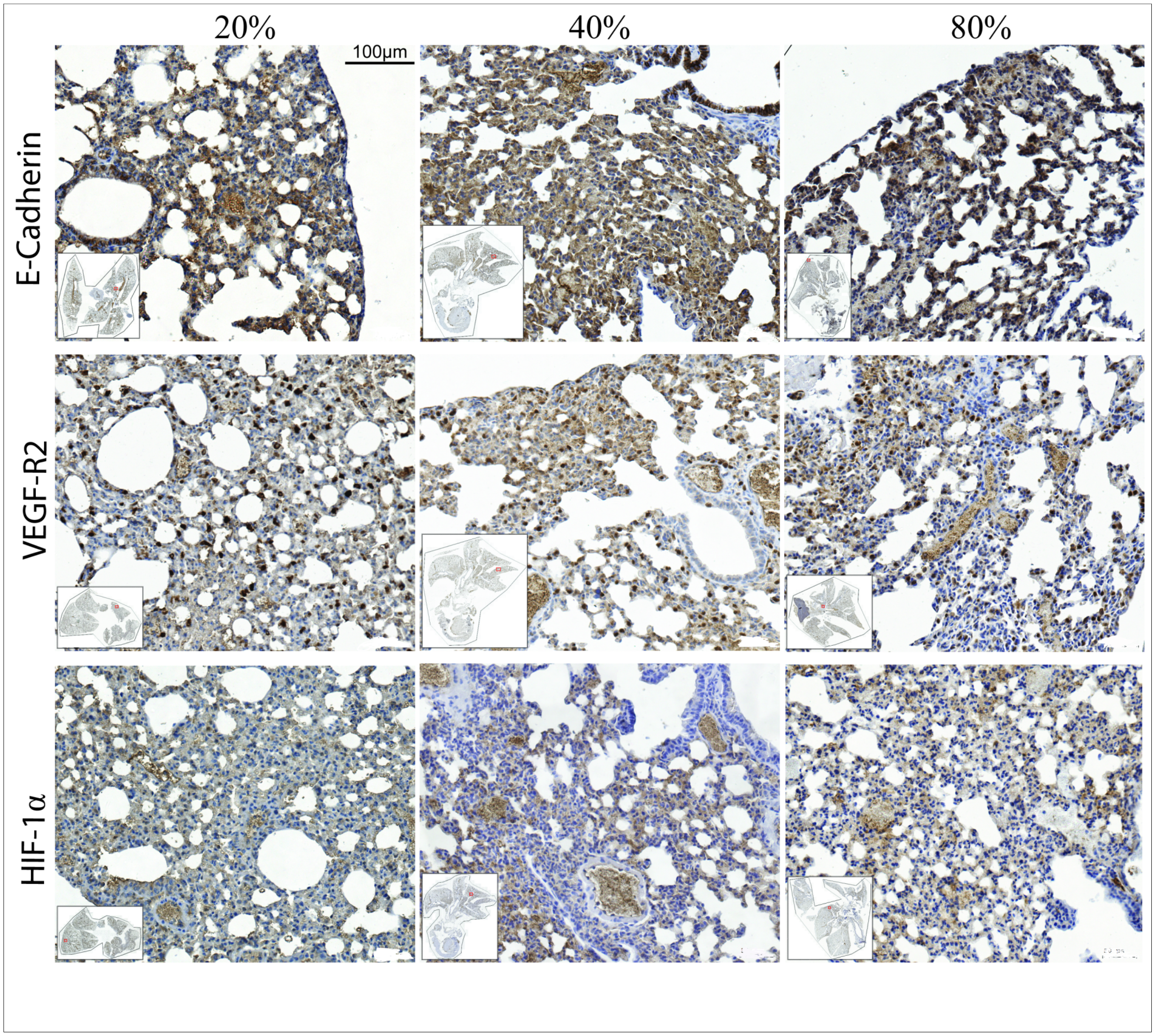

Supplement: Supplementary file 1 [file cancers-12-00688-s001.pdf]
